# Suggestion and Its Therapeutic Uses

**Published:** 1894-07

**Authors:** Samuel C. Benedict

**Affiliations:** Athens, Ga., Vice-President National Association of Railway Surgeons, Professor of Medical Jurisprudence, University of Georgia


ATLANTA
### MEDICAL AND SURGICAL JOURNAL.

VOL. XI. JULY, 1894. No. 5.

EDITED BY
LUTHER B. GRANDY, M.D., AND MILLER B. HUTCHINS, M.D.,
WITH THE CO-OPERATION OF
H. V. M. MILLER, M.D., LL.D., VIRGIL O. HARDON, M.D.,
FLOYD W. McRAE, M.D., ARTHUR G. HOBBS, M.D.,
AND H. R. SLACK, M.D., PH.M.

### *ORIGINAL COMMUNICATIONS.*

### SUGGESTION AND ITS THERAPEUTIC USES.*

BY SAMUEL C. BENEDICT, M.D., ATHENS, GA.,

Vice-President National Association of Railway Surgeons, Professor of Medical Jurisprudence, University of Georgia.

The duty devolving upon me of providing something for your annual oration was one which, at the first although inclined to shirk, I had not the bravery to, and yet the task of selection has not been an easy one.

As many of you well know, I am no orator, and I can but imperfectly deliver what I have to say to an audience, even be that audience from among mine own profession. Bear with me, therefore, to the utmost of your patience, and if the subject which I have chosen as my text should prove to be poorly handled and tediously presented, remember that my ability for this sort of work has never been recognized by any one, not even by myself, as the suggestion of a possibility.

*Orator's Address before the Georgia Medical Association, April, 1894.

The subject of physiological psychology has of late years interested me much; the study of the localization of cerebral functions is of interest to us all, but until of late years the line of thought and of study has been directed to the motor and sensory functions, and not to the higher ones of memory, reason and the emotions.

Recently, however, much thought and research have been given to the subject of the possibilities of the mind under the influence of genuine hypnosis. These researches have been made by conscientious and intelligent men, hard workers in our profession—men of undoubted worth and ability, some of whom have written treatises embodying their experiments and views.

A few years ago there was presented to this body a paper on the "Duality of the Mind," and which embodied the views of a certain school. This paper offered a topic for consideration which could not be discussed, and was not, because those of the Association present had not at that time given the subject sufficient thought. Since then a great deal has been done in this field, and the mental function of the brain, when a portion of it has been inhibited by hypnotic influence, has been studied in a scientific manner. Opportunities for fraud and deception, for collusion for pecuniary gain, of course, have large scope, but in the large majority of cases recently experimented upon, *for the knowledge to be obtained,* and *not* for money, it has been shown conclusively that there is opening up before us a field of research into the emotional and intellectual portion of man's brain which, in point of interest to us as students, and possibly in the future as practitioners, will take a front rank. It is not my intention to enter into an abstract discussion of the various conditions of hypnotism, mesmerism, clairvoyance, clairaudience, telepathy, or any of the kindred phenomena, but to take the results upon persons under the influences producing these phenomena, and to open up, if possible, with my limited observation and study of such conditions, a line of thought for some of you.

Within the past year a theory as to a law which governs psychic phenomena has appeared, the basis of the argument being the duality of the mind, not *anatomically* but functionally, although even this latter term is not exactly the one to convey the idea as to the line of argument. No theory up to this time had ever been advanced which embraced all psychological phenomena, some explaining apparently individual phenomena, but failing when confronted with another class. The schools of Mesmer and of Nancy both had a theory to explain the various phenomena, and the causes for the evolving of such phenomena, but neither could cover the whole field of clairvoyance, thought-transference or mind-reading, and yet the ablest scientists of the Royal Academy of France, after six years of patient investigation, reported that it *had* demonstrated that such powers do exist in the human mind. There is a class of phenomena about which a great variety of opinion prevails, and a great deal of skepticism, that of mental therapeutics. Take, for instance, the invocations of the gods by the Egyptian priests, by which most remarkable "cures" were made, or the magic formulæ of the disciples of Esculapius, or the healing power over certain diseases by the modern systems of Christian science, the faith and mind cures. Marvelous results are constantly being obtained through these agencies. There has until recently been no explanation offered as to how these cures are accomplished; that they *are* is not denied by many of our profession who have witnessed them. We who have never seen them are skeptical and inclined to scoff at such possibilities, attributing them to deception, or to impressions made upon nervous systems, hyperesthetic and hysterical. Do not we see constantly in our practice evidences of our influence over some of our patients by the simple power we have in making them *believe* in us? Have we not often heard it said by a patient that "All I need is to see my doctor and I get well"? "Confidence in the doctor is half the cure" is a trite saying.

Professor Bernheim's work on "Suggestive Therapeutics" is full of evidences that under certain conditions the mind of man is under absolute control by suggestion, and that it is *this suggestion* which has the power of producing the marvelous cures recorded under the writings of the various systems. Hypnotism is the state in which the person is placed when suggestion is most powerful, but in the workings of other schools cures are made without the hypnotic influence. "Faith" is a term frequently used to name the necessary mental condition for cures, and this is the term used in the Bible to express the requisite for the healings there recorded.

By suggestion is meant simply an *impression* made from without through one or more of the five senses upon the cells of the cerebral cortex; it is a sensory impression by which either motor phenomena *only* are developed, or mental action, such as a revival of memory. In some instances a language used in childhood and long since forgotten has been brought again into speech as clearly and perfectly as though never lost. *Auto-suggestion* arises without external impressions, and is typical in dreams and delirium. Now, as explanatory of many such phenomena, a working hypothesis has been brought out by Hudson, which explains better than any previous theory the cause of such phenomena. His reasoning is that man has two minds, one of which he calls the "objective" and one the "subjective mind"; and further, that this subjective mind is constantly amenable to control by suggestion and is incapable of *inductive* reasoning.

The idea of a dual mental organization is not a new one, the truth of the proposition being shown in the use by the Greek philosophers of the expression, "body, soul and spirit," and indeed the same belief is shown by all ancient philosophers, whether Christian or pagan. In recent years this doctrine is beginning to be more clearly defined, and a late work by Du Prel, based on experiments in Milan, demonstrates beyond question this dual theory by his reference to the phenomena of dreams.

The line of demarcation between the two minds has never been clearly defined until within the past year, when Hudson brings forth his theory of the objective and subjective minds. The difference between the two minds seems to be this: the *objective* mind is that portion which has the use of the five senses to take cognizance of the objective world; its impressions are received by itself and conveyed to the subjective function. This objective mind is the outgrowth of man's necessities and is his guide in maintaining his mundane existence. Its highest function is that of reasoning, in drawing conclusion inductively. The subjective mind, on the other hand, is the seat of the emotions and the storehouse of the memory, and *its* functions are under the control of the senses, or objective function. When these senses are inhibited, as by hypnosis or by sleep, the highest functions of the subjective mind are performed and most wonderful feats are witnessed. That these feats are *not* fraudulent is abundantly proven by the statements of such men as Charcot, Heidenham, Bernheim and others, as well as by the London Society for Psychological Research. As remarkable as the statement may appear, the subjective mind reads, without the organs of vision, the contents of sealed envelopes and of closed books. It appears to be that the real difference between the two minds is that the "objective" is merely the function of the physical brain, while the "subjective" is a distinct entity, possessing independent functions, capable of existing independently of the body, or in other words it is the *soul of man*. It is hard for us to reason to ourselves that such a proposition is at all tenable, especially so if no study or thought has been directed to this line of argument. At first it seems preposterous to attempt to maintain such an hypothesis, but the more you will look into this subject the more fascinating and plausible will it become. As a means to the study of mental phenomena, it is far ahead of all former theories in its possibilities for our enlightenment, and the assumption that there *is* a dual mental organization furnishes us with a foundation for our future work in dealing with mankind.

Unconsciously to ourselves we are always causing mental action in others by our suggestions, and we ourselves, by suggestions received from without, "live and move and have our being," while our auto-suggestions furnish us our dreams. In the insane mind, where the objective mind has lost its control over the subjective, auto-suggestion brings into play all sorts of visions and fancies, even to the belief that the person is some other than himself, or that he is being pursued by another whose intent is to inflict personal harm. Hallucinations, delusions and illusions are the outcome of subjective function with a lost objective control.

Whether or not the distinction between these two qualities of what we call our mind is a well defined one, it is impossible to affirm, but the operations of the mind under the influence of hypnotism has led even unbelievers to feel that there are reasons for such a demarcation. I think it is necessary to speak farther of some of these differences as observed in order to lead up connectedly to the effects of suggestion upon the mental organization.

One of the most important distinctions claimed pertains to the reasoning powers, the objective mind seeming to be able to reason inductively, deductively and analytically, while the subjective mind, when not inhibited by the objective, is incapable of any but *deductive* reasoning. A premise, no matter how false or absurd, is, when suggested, assumed as correct, and deductions of marvelous accuracy and logic evolved. Every conclusion will be so clearly and logically deduced from the major premise, and so consistently, that the listener will almost overlook the fact that the premise is assumed. Again, the most striking peculiarity of the subjective entity is its perfect memory. Modern psychology tells us that the memory of impressions made through either of the senses is recorded in the cells of the cortex, belonging to the localized area of that particular sense, and that that impression is *ineffaceable*, never obliterated, except by disease or injury, which destroys the cells of that particular area. So it is claimed by many students of psychic phenomena that while the objective mind is in perfectly normal function, most of these memory impressions are in oblivion as far as conscious cerebration is concerned, still, whereas, in delirium, trance, hypnosis, or in natural sleep, the objective mind is inhibited, the memory impressions of these cells, no matter how old or unforgotten those impressions, are again accessible to use by the subjective entity. For example, cases are on record where the extinct memory of whole languages has been suddenly restored, or, what is even more remarkable, passages in unknown tongues, learned in childhood but lost as to the then conscious memory, have been accurately recalled and repeated. This memory of the subjective mind seems to be the only kind of memory which is absolute—the ordinary memory, that of the objective mind, being more properly a recollection. A *recollection* is defined as a successful endeavor of the mind to bring into view an idea or an impression, while a *remembrance* is when the idea occurs without this endeavor, or without the external impression of the object.

Our mental development is the result of a continued series of impressions made through one or other of five senses; our cerebral cortex is the sensitive plate of the photographer and the picture of our developed mental life will be beautiful and perfect, or repulsive and unfinished according as to what kind of impressions our mental negative has received. An impression made upon any of the senses is a suggestion, and by suggestions we act and live. The action as a result of these suggestions we call reflexes. All secretion, excretion, motion is the result of reflex function, and this phenomena of life, or more exactly the cause of this phenomena, we call the "Vital Force," a term vague and indefinite. A suggestion made to us by the sight of an object, the voice of another or an impression upon the tactile sense, causes us to act or not, according as to whether our reason, the objective mind, approves or disapproves. If the reason disapproves of action upon the suggestion, it is not followed, and the suggestion to the mind that such action is not proper or desirable becomes an *adverse* suggestion, and the action of the subjective mind is inhibited. And yet, let the objective mind of man be in abeyance, as under hypnotism; let the control over the reason be inhibited, and the suggestion is received as proper and truthful and deductive reasoning follows. This has been interestingly shown by directing the course of the talk in persons who are dreaming. The line of thought in the dream has been followed out and kept up by suggestion from one awake and intelligent answers given, and yet the action of the mind is *below* the threshold of consciousness to the dreamer, for upon awakening he has no remembrance of his dreams of suggestion or of auto-suggestion. The same is true in the hypnotic state, when deductive reasoning from a suggestion can be carried on for a long time without any consciousness on the part of the person hypnotized. The subjective mind is then amenable to control by suggestion.

Now, if the functional activity of our organs is under the control of reflexes upon the cerebral cortex, and suggestion, under favorable circumstances, can exercise control upon this same cortex, why may not suggestion act favorably in the relief of *abnormal* function? It is claimed by hyponists that this can be done, and has been done. Perfect anesthesia to pain has been brought about by hypnosis, and hundreds of persons have had surgical operations performed upon them without pain, just as our chemical anesthetics may suspend the perception of pain by the cortex. Charcot has demonstrated beyond question, that all the symptoms of "railway spine" may be produced by suggestion upon hypnotized subjects. The hypochondriac, by continued introspection, develops a pain or a disease by his auto-suggestion. It is claimed by Hudson that the functions, conditions and sensations of the body are independent of the objective mind, and are under the absolute control of the subjective, and that under favorable conditions many functional diseases or abnormal sensations have been removed by the suggestion that they do not exist.

It is a well known fact that the *symptoms* of almost any disease can be produced in hypnotic subjects by the power of suggestion; fever, as shown by the thermometer, has been produced, partial and total paralysis, flushed faces and chills, and Professor Bernheim, in his "Suggestive Therapeutics," records the case where he produced a blister upon the back by suggesting that a postage stamp was the blister applied. But as absolutely necessary to the success of any relief by suggestion, the person must have entire and implicit faith in the ability of the operator to do as he claims, for otherwise an *adverse* suggestion is aroused in the objective mind, and then no effect upon the subjective can be produced. Have we not in our own professional experiences found persons, who, having perfect confidence in our ability, were relieved of symptoms of a disease by our statement that none existed? Or, on the other hand, a hypochondriac made the more miserable by the suggestion of a new symptom in his disease? Do not our placeboes act by suggestion? Are not our bread pllls an evidence that the relief is obtained either in due course of nature's reparative process, or by the impression made upon the subjective mind by the suggestion that they are all that is necessary to effect a cure?

As physiological students we understand the control of the organic functions of the body to lie in the cells of the cerebral cortex. We now know that temperature centers, pain centers and probably trophic centers exists. Stimulation to secretion or excretion is produced by peripheral irritation through afferant nerve fibers to the cells of spinal ganglia, or the cerebral cells; drugs, acting as peripheral or as central irritants, increase or decrease such functions, while mental impressions, even auto-impressions, produce the same. The thought or auto-suggestion of food, or of a pleasure, often produces the desire for such. If, therefore, cells which receive afferent impulses are also influenced by intra-cranial sensations, why may not suggestion to these same centers influence them also? It is claimed, and probably justly so, that there resides in man a psychic power over the sensations and functions of the body, and that this power can be evoked by will, under certain conditions other than by hypnosis, to the alleviation of human suffering. There is no doubt in my own mind of the truth of the assertion. I am not now speaking of diseases or their symptoms due to organic parenchymatous changes, but to functional disturbances of the nervous system, and of the result of such disturbances upon organic function. Many examples of healing by suggestion are recorded in both the Old and New Testaments; they are full of such examples.

In modern times marvelous cures are effected through these means, no matter what the *modus operandi* of the particular school may be. The thousands of cures at the famous grotto of *Lourdes* by *faith* in its healing power are unquestioned to-day, and who will deny but that some of the "cures" of our own "Hillsmanshaft" are due to suggestion? Medical history records the case of a man with a paralyzed tongue who was made to believe in the power of a certain instrument, unknown to him, to cure his malady. Previous to the use of the instrument a clinical thermometer was placed in his mouth for the purpose of taking his temperature, when he, believing it to be the wonderful instrument, cried out that he could move his tongue freely. The cure of an English lady of a chronic case of a tooth-bristle in the throat, by the removal of a bristle previously placed in the forceps, is a well known instance of the power of suggestion.

The schools of mind cure and of Christian science furnish examples of cures by suggestion, the methods of obtaining the "faith" necessary to its successful use being different.

In the pursuit of our vocation as physicians we have to deal with a generation of people who have an hereditary belief in the power of medicines to "cure" all diseases. Many of us use medicines as though we, also, shared the same belief, and yet there is no doubt but that the majority of us are skeptical as to this power in remedies, and we use many simply as placeboes. Often is it the case that a patient, believing that his physician has the power by the use of a remedy to cure his disease, will get well by the effect of this suggestion to his subjective mind. Can this proposition be successfully denied?

According to the theory of Hudson all diseases are curable by mental processes, but practically the field is very limited. The boundaries are not well defined, as much depends upon the idiosyncrasies and environment of the patient. The success of the physician is as largely dependent upon his knowledge of these conditions as upon his diagnosis and medical treatment, and the success of the purely mental healer depends largely upon his knowledge of the patient's habits of thought, his beliefs, prejudices and mental environment.

In this short review of the present status of psychological literature I do not dare to hope that I have presented a sufficiently well connected line of thought to give you a clear line of demarcation between the objective and subjective minds. I realize that I am incompetent so to do, but if the suggestion to your minds that such a duality *does* exist will cause you to further look into this very interesting subject, I shall be satisfied. A few words then, in conclusion, as to the possibilities from the use of suggestion in our every-day professional work.

Assume, if you please, the theory of Hudson of the duality of the functional mind, and by looking over your past experiences with patients see if you do not find a potent factor in your power to impress the mind of a patient that he can be cured of his functional disorder, and *is*, by the suggestion to his confiding mind that what you do will relieve him. And right here the power of the person's own auto-suggestion, his belief, comes into use. The successful workers in the field of Christian science are those who so educate their patients and pupils to help themselves, that their confidence in themselves acts through auto-suggestion upon the subjective mind, so as to keep in normal control the functions. How often in our practice have we heard it said, or known it to occur, in epidemics that the fearless escape the disease. The belief (or auto-suggestion) that they are proof against the contagion proves a sufficient power upon subjective function-control to avoid greater resisting power than is found in the timid and nervous. We assure a patient that this or that symptom will disappear under the use of a certain remedy, and it does so, and yet does that necessarily prove that the remedy by its inherent virtue produces cure? Will not bread pills often cure the sick? Why is it that we so often see the course of a functional disease either entirely relieved or made worse, according as to whether the suggestion to the mind is one of joy or one of worry and sorrow? The functional activity of our organs is certainly under the control of intracranial cells. Grief, worry, care or anxiety will disturb the normal action of these organs, whilst joy, pleasure or desires obtained will relieve in many instances functional disorders. The simple suggestion or assurance to our patient that a particular unpleasant symptom "means nothing" is often all-sufficient to cause the impression of that symptom to disappear. On the other hand, if a patient has "faith" in our judgment and skill, the statement that a certain intercostal pain means heart, stomach, liver or lung disease will often be all that is necessary for him to develop, as far as he knows them, the actual symptoms of such disease. Such examples in our every-day life could be multiplied *ad infinitum*. Humphreys specifies act by suggestion, or they act not at all. The proposition of Hudson is, therefore, the more readily accepted by the medical man than by the layman, no matter whether we accept his dual theory of the objective and subjective function, or simply call both "the mind."

To repeat once more the proposition is this:

1. The subjective mind exercises complete control over the functions and sensations of the body.

2. The subjective mind is constantly amenable to control by suggestion to the objective mind; and

3. Consequently, the functions and sensations of the body can be controlled by suggestions to the objective mind.

The gist of the above propositions is that by our will power we may control the condition of our organs as to functional activity.

If in the foregoing discussion I have taxed your patience too far by suggesting to you a line of thought which appears unreasonable, unscientific, or absurd, pardon me, gentlemen, if I have no other excuse to offer you than my belief that if you will only look into this interesting subject as I have done, with an unbiased mind and with the desire to gain knowledge of this subject as an honest investigator in this psychic field, you will appreciate my interest in the question and look leniently upon my humble effort to furnish you with food for thought differing from the ordinary menu.

106 *E. Clayton Street.*

